# P-1024. Fungal Pneumonia and Severe Neutropenia: Risk Factors for Increased 90 Day Mortality

**DOI:** 10.1093/ofid/ofae631.1214

**Published:** 2025-01-29

**Authors:** Guy Handley, John Greene, Anthony P Cannella, Ana Velez, Shivan Shah, Ju Hee Katzman, Yanina Pasikhova

**Affiliations:** University of South Florida Morsani College of Medicine, Tampa, Florida; Moffitt Cancer Center, Tampa, FL; Moffitt Cancer Center and University of South Florida Morsani College of Medicine, Tampa, Florida; University of South Florida, Tampa, Florida; University of South Florida, Tampa, Florida; University of South Florida / Moffitt Cancer Center, Tampa, Florida; Moffitt Cancer Center, Tampa, FL

## Abstract

**Background:**

Invasive fungal infections (IFI) carry a high mortality risk in hematologic malignancy; the majority being pulmonary infections. Diagnosis is based on consensus definitions of host factors, clinical features and mycologic evidence. In previous studies, increased mortality is associated with delays in therapy and failure of neutrophil recovery; however, use of salvage posaconazole may have improved outcomes. We sought to identify risk factors associated with higher 90-day mortality in these patients.

**Figure:**
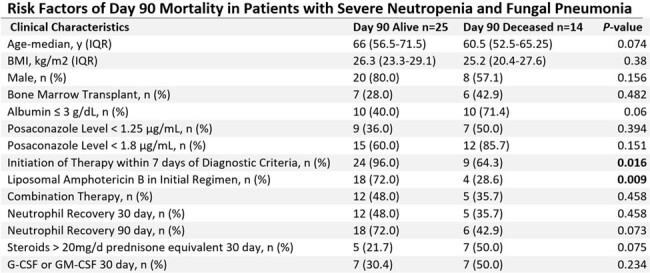

**Methods:**

Patients at an NCI designated comprehensive cancer center from 06/2021 – 07/2023 with hematologic malignancy, severe neutropenia and a diagnosis of IFI and receiving posaconazole for treatment with verified therapeutic drug monitoring were identified. Demographic, clinical and laboratory data were evaluated to identify risk factors for increased mortality within 90 days of diagnosis of pulmonary IFI. The diagnosis was based on international consensus definitions.

**Results:**

A total of 39 patients were identified. 18 (46.2%) were diagnosed with acute myeloid leukemia and 13 (33.3%) had undergone bone marrow transplantation. Median time from diagnosis to treatment initiation was 2 days and 17 (43.6%) patients received combination therapy with multiple antifungal agents. Bronchoscopy with bronchoalveolar lavage was performed in 34 (87.2%) and of those a microbiologic diagnosis found in 5 (14.7%). Day 90 mortality was 56.0% (n=14). Survivors were more likely to have initiation of antifungal therapy within 7 days (96.0% vs 64.3%, p=0.016) and received liposomal amphotericin B as part of their initial therapy (72.0% vs 42.9%, p=0.009). Patients receiving corticosteroids or who failed to have neutrophil recovery tended to have higher mortality, but this did not achieve statistical significance. Combination therapy and receipt of granulocyte or granulocyte-macrophage colony-stimulating factors were not associated with improved day 90 mortality.

**Conclusion:**

Early recognition and initiation of antifungal therapy in high-risk neutropenic patients with hematologic malignancy with IFI and initial therapeutic regimens including liposomal amphotericin B may be associated with improved outcomes.

**Disclosures:**

**All Authors**: No reported disclosures

